# FXYD2 mRNA expression represents a new independent factor that affects survival of glioma patients and predicts chemosensitivity of patients to temozolomide

**DOI:** 10.1186/s12883-021-02476-2

**Published:** 2021-11-09

**Authors:** Kaijia Zhou, Tao Jiang, Yanwei Liu, Zheng Zhao, Lijie Huang, Guanzhang Li

**Affiliations:** 1 Neuro-Oncology Surgery Department of Fujian Cancer Hospital & Fujian Medical University Cancer Hospital, Fuzhou, 350014 China; 2 Beijing Neurosurgical Institute, Capital Medical University, Beijing, 100070 China; 3 Department of Neurosurgery, Beijing Tiantan Hospital, Capital Medical University, Beijing, 100070 China; 4 Center of Brain Tumor, Beijing Institute for Brain Disorders, Beijing, 100070 China; 5 China National Clinical Research Center for Neurological Diseases, Beijing, 100070 China

**Keywords:** FXYD2, Glioma, Chemotherapy, Prognosis, Molecular profile

## Abstract

**Purpose:**

Glioma is the most common primary intracranial tumor. Owing to the poor prognosis associated with high-grade gliomas, there is an urgent need to identify biomarkers related to prognosis and treatment sensitivity. Here, we analyze the expression of FXYD2 mRNA in gliomas, and explore its clinical prognostic value and significance in this disease.

**Methods:**

Clinical features, FXYD2 mRNA expression levels, and survival data were analyzed for 516 glioma patients from the Chinese Glioma Genome Map Project, 481 from the cancer genome map datbase and 268 from the molecular braintumor database. The expression patterns for FXYD2 mRNA were compared using the chi-square test, and overall survival (OS) of glioma patients was evaluated according to FXYD2 mRNA expression levels. The factors affecting glioma survival were evaluated by Cox univariate and multivariate regression analysis.

**Results:**

FXYD2 mRNA expression was related to the grade of gliomas. The higher the level, the lower the expression. Meanwhile related to the pathological classification of gliomas. Oligodendroglioma, IDH-mutant and 1p/19q-codeleted was higher than Astrocytoma, IDH-mutant, higher than Glioblastoma, IDH-wildtype. Moreover, temozolomide (TMZ) chemotherapy was found to be an independent factor affecting survival in patients with high FXYD2 mRNA expression, but not in patients with low expression.

**Conclusion:**

FXYD2 mRNA expression represents a new independent factor affecting the survival of glioma patients and may serve as an independent prognostic indicator to predict the sensitivity of gliomas to TMZ.

## Introduction

Gliomas, the most common primary malignant tumor of the brain [1], was classified in 2021 by the World Health Organization (WHO) based on histopathology, isocitrate dehydrogenase (IDH) mutation and 1p19q co-deletion [2]. Moreover, the prognosis of high-grade gliomas remains poor, particularly in patients with glioblastoma who have a 5-year survival rate of only 5% [1, 3], even after administration of the standard three treatments: maximum surgical resection, radiotherapy, and chemotherapy. In fact, the average survival time for glioblastoma patients is only 14 months [4]. Due to the heterogeneity of gliomas [5] and different drug resistance to temozolomide (TMZ) [6–9], the prognosis of different glioma patients is quite different. Meanwhile, the application of high-throughput technology for the molecular classification of gliomas as well as for screening differentially expressed genes and drug resistance genes has become a research hotspot to facilitate the development of corresponding targeted drugs.

Na/K-ATPase is an oligomeric transmembrane protein composed of α, β, and γ subunits that functions to maintain the dynamic membrane potential and is associated with many cellular functions as well as the pathogenesis of specific diseases [10]. Specifically, Na/K-ATPase upregulation has been reported in various cancers [11–15]. Meanwhile, inhibiting Na/K-ATPase activation and expression effectively inhibits cancer cell proliferation and survival [16, 17]. FXYD2 (sodium/potassium-transporting ATPase subunit gamma) is the γ subunit of the Na/K-ATP enzyme and functions as a regulator of the enzyme activity [18]. Interestingly, a previous study reported that in ovarian clear cell carcinoma (CCC) patients, the expression level of FXYD2 was positively correlated with patient prognosis. Specifically, upregulated FXYD2 expression increased the sensitivity of ovarian CCC cells to the Na/K-ATPase inhibitor cardiotonic glycoside, thereby enhancing its therapeutic effect. However, the expression pattern and clinical significance of FXYD2 have not yet been reported in gliomas.

Here, through transcriptome sequencing, this study sought to establish the relationship between FXYD2 mRNA expression and the clinical features and survival data for glioma cases collected from the Chinese Glioma Genome Map Project as well as TCGA and REMBRANDT databases.

## Data and methods

### Data collection

Data, including clinical information (sex, age), histopathology, WHO grade, molecular markers (*IDH* mutation, *1p/19q* deletion), and follow-up information (survival time), of 516 glioma patients were collected from the Chinese glioma Genome Map Project (CGGA). The enrolled patients were treated by craniotomy in Beijing Tiantan Hospital, China Medical University, Beijing sanbo Hospital Affiliated to Capital Medical University, the First Affiliated Hospital of Nanjing Medical University, Harbin Medical University or General Hospital of Tianjin Medical University from 2005 to 2017. Inclusion criteria were: (1) patients diagnosed with supratentorial cerebellar tumor; (2) patients > 18 years old; (3) patients diagnosed with diffuse glioma; (4) patients signed informed consent. Exclusion criteria were: (1) patients suffering other tumors than glioma (e.g. patients suffering from cerebral lymphoma of melanoma metastasis); (2) patients aged younger than 18 years; (3) patients with other malignat diseases or fixed tumors. All specimens were collected under IRB KY2013-017-01 and frozen in liquid nitrogen within 5 min after resection. All subjects were unanimously diagnosed as supratentorial diffuse gliomas according to the central pathological examination of an independent committee certified neuropathologist. All patients were classified according to the 2021 World Health Organization (WHO) classification of tumors of the central nervous system [2]. The overall survival (OS) rate of clinical end-point events was calculated from the initial pathological diagnosis to death or last follow-up. This study was approved by the Institutional Ethics Committee of Beijing Tiantan Hospital (KY2014–002-02), and complied with the principles of the Helsinki declaration. All patients provided written informed consent.

### mRNA sequencing

#### mRNA transcriptome sequencing

According to the manufacturer’s instructions, total RNA was extracted with an RNeasy Mini Kit (Qiagen). Pestle and QIAshredder (Qiagen) were used to crush and homogenate the frozen tissue. The RNA integrity was assessed via electrophoresis using the 2100 bioanalyzer (Agilent Technologies), and only high-quality samples with RNA integrity numbers (RIN) ≥ 6.8 were used to construct the sequencing library. Briefly, 1 μg of total RNA was used in conjunction with the TruSeq RNA library preparation kit (Illumina). With the exception of SuperScript III reverse transcriptase (Invitrogen) used the synthesis of the first strand of cDNA, all other operations were low-throughput. Following PCR amplification, and purification of the junction fragments, the DNA concentration of the junction was determined by quantitative PCR (biological system 7500) with QP1 5′-AATGATACGGCGACCACCGA-3′ primers and QP2 5′-CAAGCAGAAGACGGCATACGAGA-3′ primers. The length of the DNA fragment was measured using a 2100 bioanalyzer, and the median size of the inserted fragment was 200 bp. The RNA-seq library was sequenced using the Illumina HiSeq 2000 Universe 2500 Universe 4000 sequencing system. The library adopts a paired end strategy, with reading lengths of 101 bp, 125 bp, or 150 bp. Base invocation was performed using the Illumina Casava v1.8.2 pipeline.

### Mapping and quantification

STAR (v2.5.2b, Dobin et al., 2012) and RSEM (v1.2.31, Li et al., 2011) software were used for RNA-seq mapping and quantification. These reads were then compared with the Human Genome reference (GENCODE v19, hg19) for STAR, after which RSEM was used to calculate the sequencing reads for each GENCODE gene. The expression levels of different samples were combined into an FPKM matrix (fragments per million fragments per kilobase transcriptome). Only when the expression level was > 0 in half the samples was a gene defined as expressed. Finally, we retained only the expressed genes in the mRNA expression profile.

### RNA-Seq comparison workflow

STAR (v2.5.2b) was used to compare the mRNA profiles. For each RNA-seq sample, STAR compares each read group with the human reference genome (GENCODE v19, hg19), and then merges the alignment results. This workflow generates a BAM file that contains both aligned and unaligned reads (against data). All experimental methods were carried out in accordance with the relevant guidelines and regulations that were previously reported [19]. Preparation, sequencing, and data analysis of the RNA-seq library were the same as that described previously [20].

### Identification of IDH mutation and 1p/19q co-deletion

Aligned the RNA-seq sequencing reads to the human reference genome. and counted the reads position which supporting mutation at chr15:90631838 chr2:209113112 and chr2:209113113 to determinate the IDH mutation status. Gene expression was used to predict 1p/19q status. Firstly, obtained the proportional gene expression profiles of 1p and 19q genes sorted by genomic location. Then smoothed 1p and 19q expression levels using a sliding 100 gene window. Finally, used the clustering method to determine the co-deletion status of 1p and 19q.

### O6-methylguanine-DNA methyltransferase (MGMT) methylation detection

After the samples were treated with bisulfite, the promoter region was sequenced to identify the methylation sites, and the average methylation ratio of each site was calculated. According to the research results, the cut-off value of the average methylation level in the sequencing section is set to 8%: the samples with the average methylation level ≤ 8% are MGMT methylation negative, and the samples with the average methylation level > 8% are MGMT methylation positive.

### Histological types

In this study, “Histopathological types (HE)” in Table.1 means the histological and pathological types were determined based exclusively on morphological features on hematoxylin-eosin sections. While “histological types” in “2021 WHO classification” Table.1 were based on “integrated” histological and molecular features.

**Table 1:** Relationship between FXYD2 mRNA expression and clinical features in 516 glioma patients

Parameter	Variable	N	FXYD2 mRNA expression				χ2	*P* value
			Low	%	High	%		
Sex	Female	225	115	51.1	110	48.9	0.197	0.657
	Male	291	143	49.1	148	50.9		
Age^a^	< 43	238	119	50.0	119	50.0	0.000	1.000
	≥ 43	278	139	50.0	139	50.0		
Progression status	Primary	301	129	42.9	172	57.1	14.743	0.000
	Recurrent	215	129	60.0	86	40.0		
Histopathological types (HE)^b^	oligodendroglioma	114	31	27.2	83	72.8	31.937	0.000
	astrocytoma	218	117	53.7	101	46.3		
	glioblastoma	184	110	59.8	74	40.2		
IDH mutation status	Wildtype	224	155	69.2	69	30.8	58.347	0.000
	Mutant	292	103	35.3	189	64.7		
1p/19q codeletion status	Non-codel	401	231	57.6	170	42.4	41.636	0.000
	Codel	115	27	23.5	88	76.5		
MGMT methylation_status	methylated	244	115	47.1	129	52.9	0.564	0.453
	un-methylated	169	86	50.9	83	49.1		
	NA	103	57	55.3	46	44.7		
2021 WHO classification	Oligodendroglioma, IDH-mutant and 1p/19q-codeleted	114	31	27.2	83	72.8	64.092	0.000
	Astrocytoma, IDH-mutant	186	77	41.4	109	58.6		
	Glioblastoma, IDH-wildtype.	147	97	66.0	50	34.0		
	Astrocytoma, IDH-wildtype (NEC)^c^	69	53	76.8	16	23.2		
2021 WHO grade	2	134	60	44.8	74	55.2	10.951	0.004
	3	198	88	44.4	110	55.6		
	4	184	110	59.8	74	40.2		
Complete excision	No	126	61	48.4	65	51.6	0.095	0.758
	Yes	372	186	50.0	186	50.0		
	NA	18	11	61.1	7	38.9		
Radiotherapy	No	108	54	50.0	54	50.0	0.000	1.000
	Yes	408	204	50.0	204	50.0		
Chemotherapy	No	124	60	48.4	64	51.6	0.170	0.680
	Yes	392	198	50.5	194	49.5		

^a^The patients were divided into two groups according to the median age of 43 years

^b^The histological and pathological types were determined based exclusively on morphological features on hematoxylin-eosin sections

^c^NEC means that the necessary molecular markers have been successfully detected, but the results cannot be classified into the current who glioma classification

### Verification group data collection

The clinical, histopathological, and survival follow-up data, as well as FXYD2 mRNA sequencing data, for glioma patients were collected from two open independent datasets. Among them, 481 cases were from the cancer genome map database (TCGA, http://www.cgga.org.cn/download_other.jsp), and 268 were from the molecular brain tumor database (REMBRANDT, http://www.cgga.org.cn/download_other.jsp).

### Statistical analysis

R software 3.3.2 and SPSS software 25.0 were used to perform all statistical analyses and to generate box scatter plots and survival curves. The normally distributed data were expressed as mean ± standard deviation (x ± s). Student’s t-tests, one-way ANOVA, and LSD-t pairwise comparisons were used to compare FXYD2 mRNA expression in different groups. Kaplan–Meier curve and log rank test were used to analyze the OS of patients in different groups. Univariate and multivariate Cox regression analyses were used to analyze the factors affecting the survival time of glioma patients. All statistical analyses were bilateral, and results were considered statistically significant at *P* < 0.05.

## Results

### Clinical features and FXYD2 mRNA expression in 516 glioma patients in CGGA

Clinical features included sex, age, recurrence status, histopathology, WHO grade, IDH mutation status, 1p/19q co-deletion status, methylation of O6 methylguanine DNA methyltransferase (MGMT), radiotherapy history, chemotherapy, and integrated diagnosis according to 2021 WHO classification. The histopathology (HE) were determined based exclusively on morphological features on hematoxylin-eosin sections. The 516 glioma patients were then classified as having low or high FXYD2 mRNA expression based on median expression values (Table 1). The median expression value was ≥ the median expression value and < median expression value (Table 1).

### Relationship between FXYD2 mRNA expression and clinical features in glioma patients

The relationship between clinical characteristics and FXYD2 mRNA expression was analyzed. FXYD2 mRNA expression was not associated with sex (*P* = 0.657), age (*P* = 1.000), methylation of MGMT (*P* = 0.453) or radiotherapy or chemotherapy history (*P* = 1.000, *P* = 0.680, respectively) in glioma patients. It was, however, significantly correlated with recurrence (*P* = 0.000), histopathological types (HE) (*P* = 0.000), 2021 WHO grade (*P* = 0.004), *IDH* mutation (*P* = 0.000), *1p/19q* co-deletion (*P* = 0.000), and 2021 WHO classification (*P* = 0.000) (Table 1).

### FXYD2 MRNA expression is higher in glioma patients with better prognosis

**Fig. 1:**
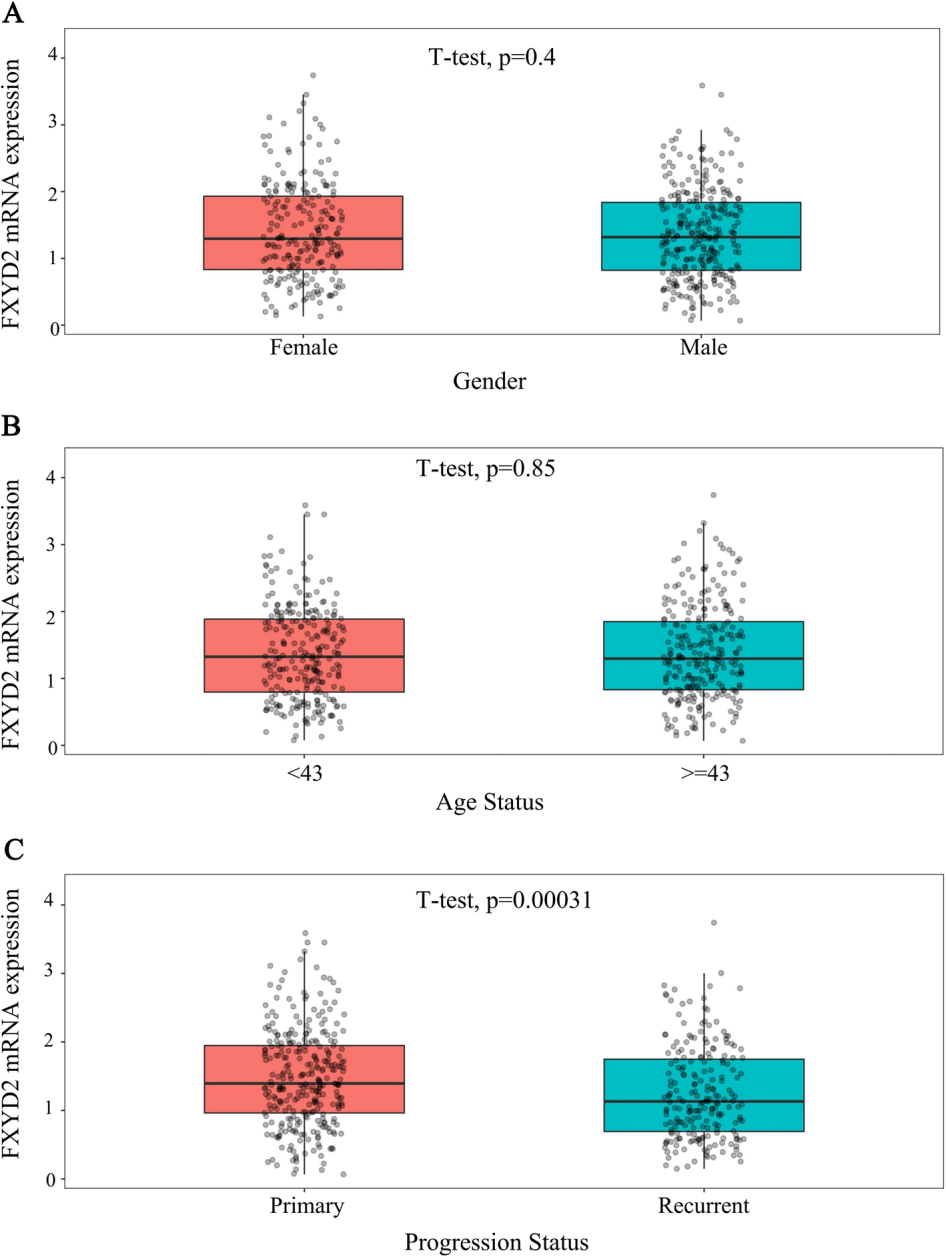
The expression of FXYD2 mRNA in different gender, age and recurrence status of gliomas in CGGA. The expression of FXYD2 mRNA in 516 glioma patients with different gender (**A**), age (**B**) and recurrence status (**C**) was compared by student’s t-test. Data represent mean ± SD

**Fig. 2:**
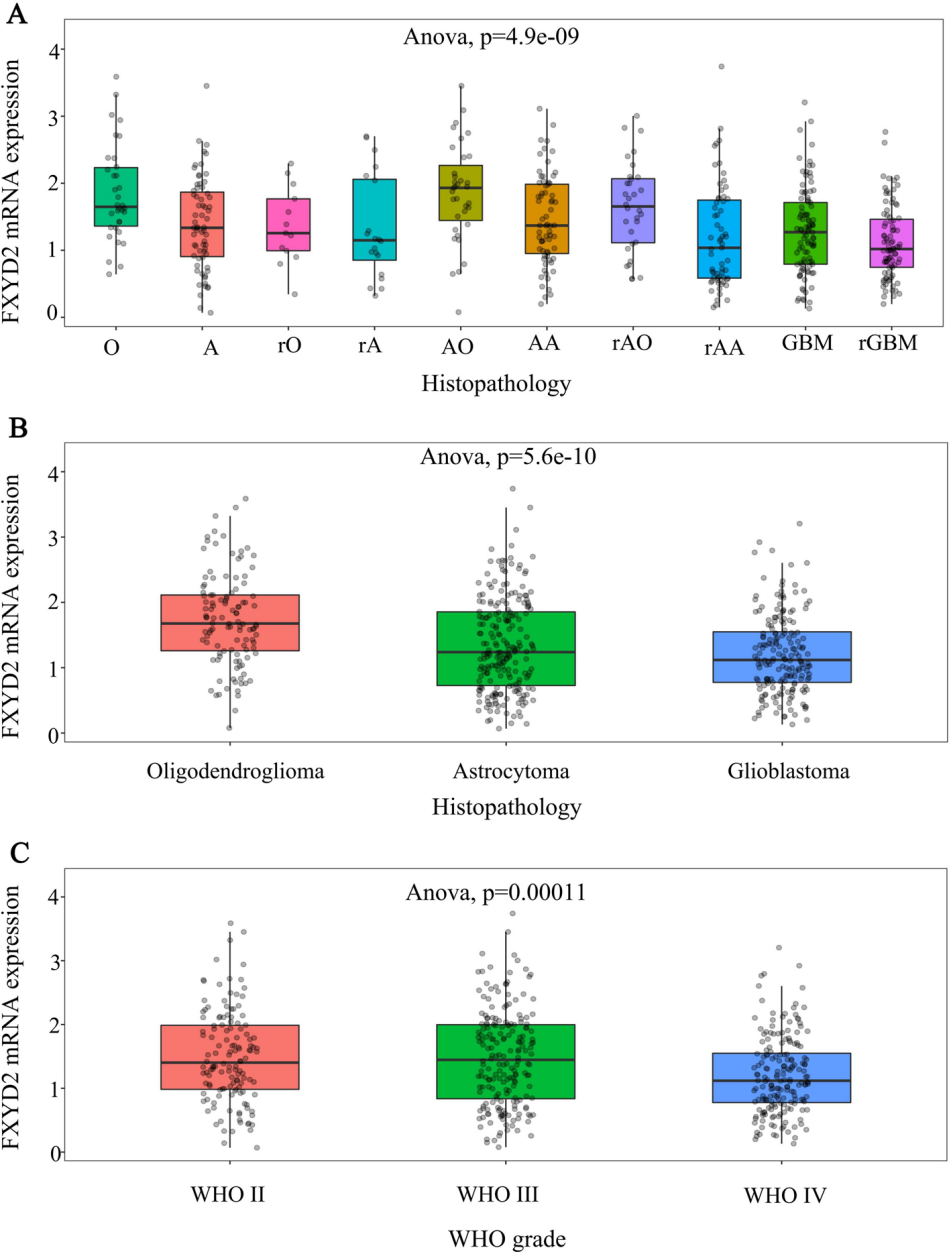
The expression of FXYD2 mRNA in different histopathology and WHO grade in CGGA. The expression of FXYD2 mRNA in 516 glioma patients with different histopathology (**A**, **B**) and WHO grade (**C**) was compared by ANOVA. Data represent mean ± SD

**Fig. 3:**
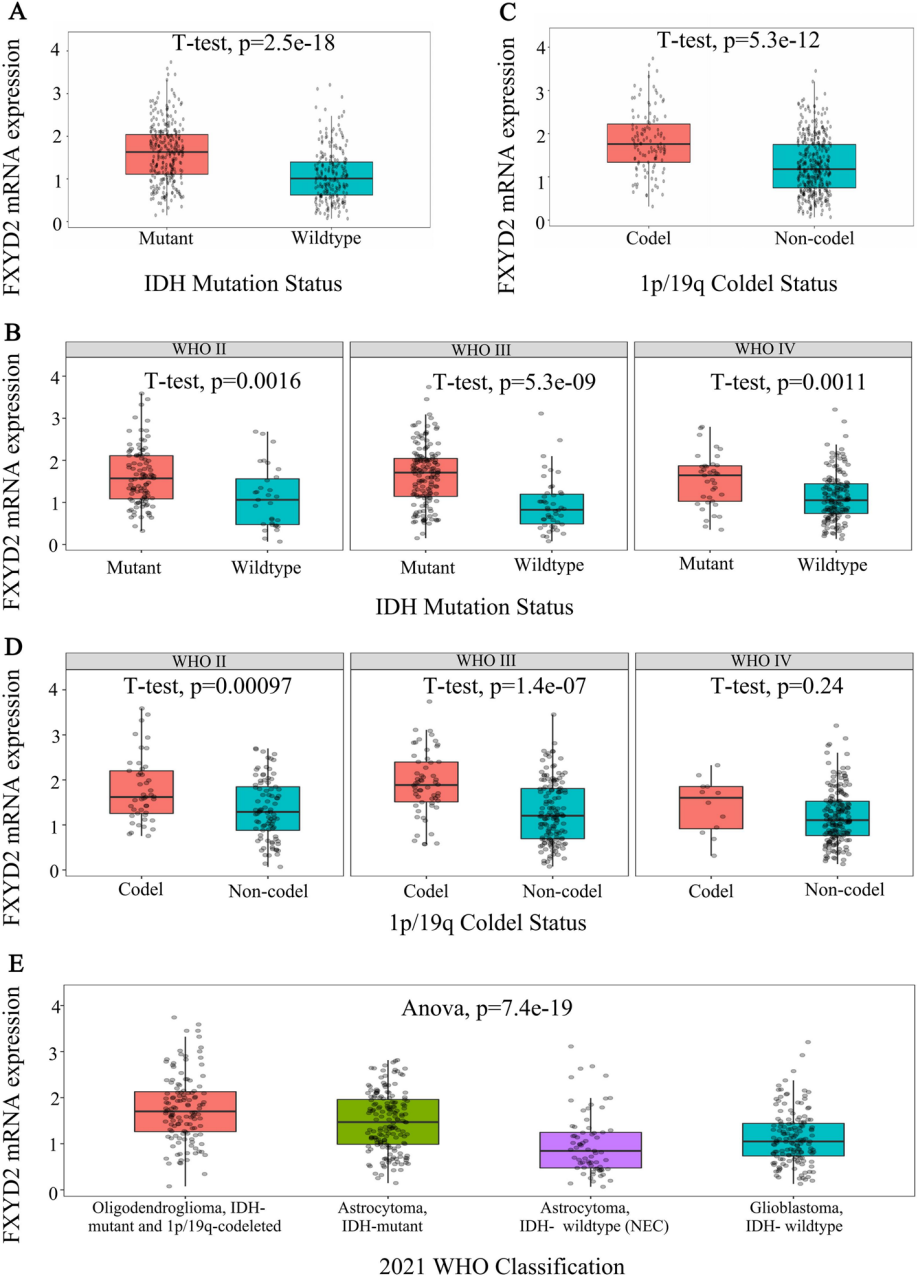
The expression of FXYD2 mRNA in different IDH mutation and 1p/19q col-deletion status in CGGA. The expression of FXYD2 mRNA in 516 glioma patients with different IDH mutation status (**A**) and different 1p/19q deletion status (**C**) was compared by student’s t-test. The expression of FXYD2 mRNA in patients with different IDH mutation status (**B**) and 1p/19q deletion status (**D**) was compared by student’s t-test in different WHO grades of glioma patients. There were 134 cases of WHOII, 198 cases of WHOIII and 184 cases of WHOIV. The expression of FXYD2 mRNA (**E**) in 516 glioma patients with different WHO classification was compared by ANOVA. Data represent mean ± SD

FXYD2 mRNA expression in glioma patients with different clinical and molecular pathological features was compared using a scatter plot. The clinical features assessed included sex, age, recurrence (Fig. 1), histopathology (HE), WHO grade (Fig. 2), *IDH* mutation, *1p/19q* co-deletion status, and 2021 WHO classification (Fig. 3). The results showed that the expression of FXYD2 mRNA was higher in patients with a good prognosis, including those with primary glioma (*P* = 0.00031), oligodendroglioma (*P* = 5.6e-10), lower WHO grade (*P* = 0.00011), *IDH* mutation (*P* = 2.5e-18), *1p/19q* co-deletion (*P* = 5.3e-12), and Oligodendroglioma, IDH-mutant and 1p/19q-codeleted (*P* = 2.3e-20).

**Fig. 4:**
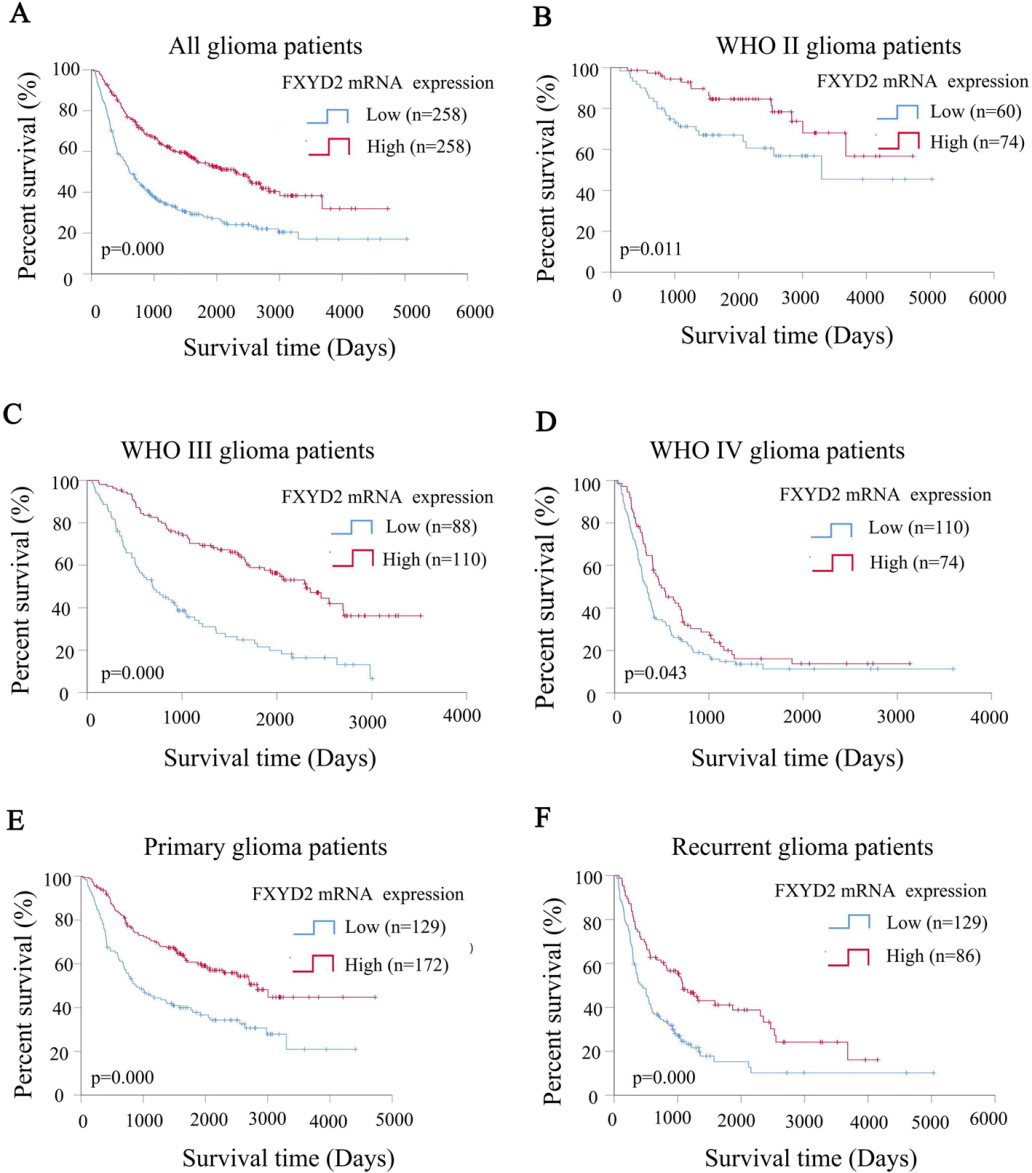
Kaplan–Meier curves for overall survival (OS) of different FXYD2 mRNA expression in 516 glioma patients with different WHO grades and different recurrent status in CGGA. The Kaplan–Meier curves for overall survival (**A**-**D**) of different FXYD2 mRNA expression in glioma patients with different WHO grades. The Kaplan–Meier curves for overall survival (**E**, **F**) of FXYD2 mRNA expression in patients with different recurrent gliomas. Log rank test was used to compare the difference between the two survival curves

Moreover, the survival time for glioma patients with high FXYD2 mRNA expression was longer. Kaplan–Meier survival curves were used to explore the effect of FXYD2 mRNA expression on the total survival time of glioma patients. The results show that the survival time of patients with high expression of FXYD2 mRNA was longer than that of patients with low expression of WHOII (*P* = 0.000; Fig. 4A). After stratifying the data according to WHO grade, the same result was observed in patients with all grades of glioma: WHOII (*P* = 0.011; Fig. 4B), WHOIII (*P* = 0.000; Fig. 4C), and WHOIV (*P* = 0.043; Fig. 4D). The same results were also obtained for patients with primary initial gliomas (*P* = 0.000; Fig. 4E) and relapse (*P* = 0.000; Fig. 4F).

### FXYD2 mRNA expression can predict the survival and prognosis of glioma patients

**Fig. 5:**
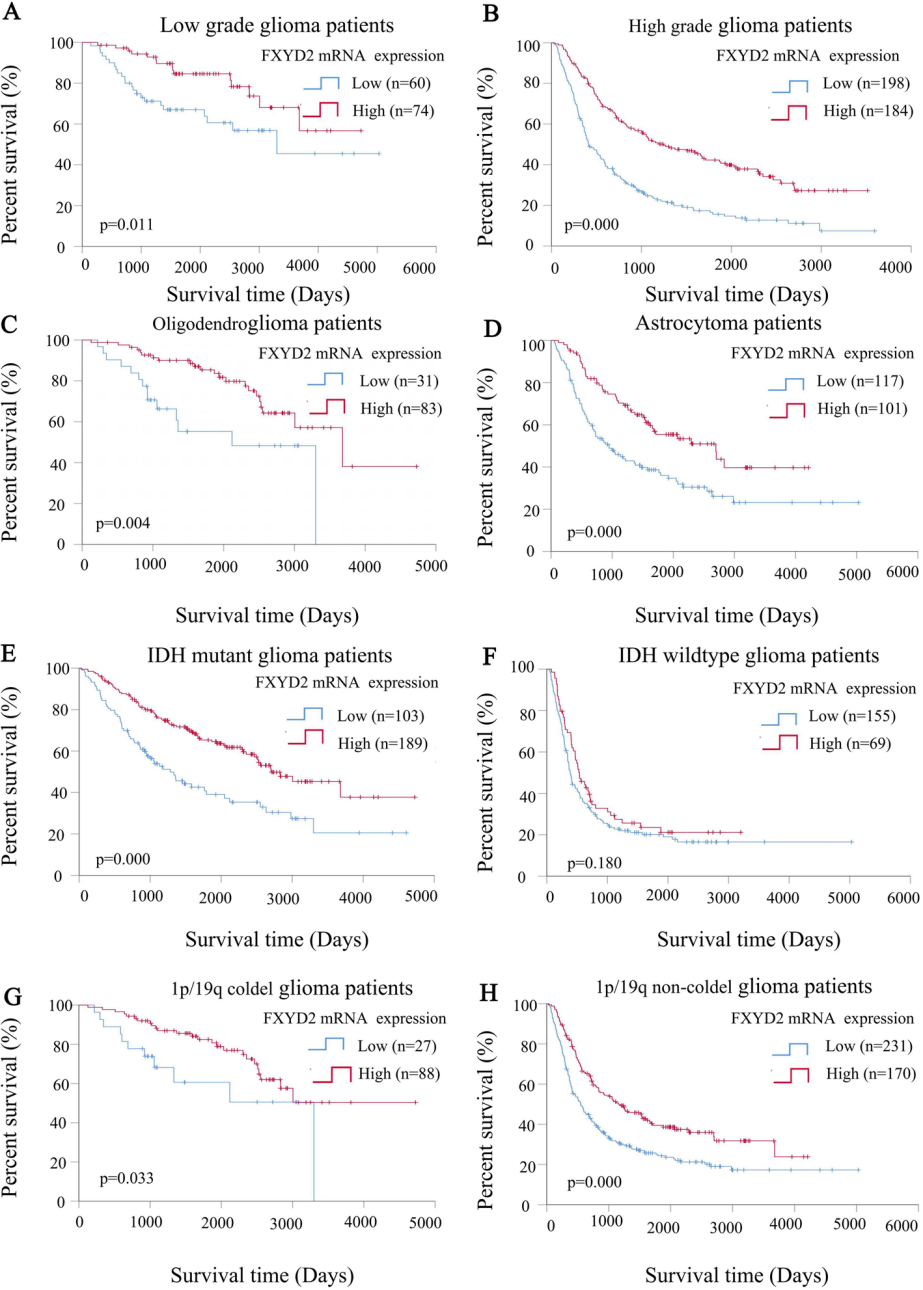
Kaplan–Meier curves for overall survival of different FXYD2 mRNA expression in CGGA glioma patients with different histologic type, WHO classification, IDH mutation and 1p/19q col-deletion status. The Kaplan–Meier curves for overall survival of different FXYD2 mRNA expression in glioma patients with different WHO grade (**A**, **B**), histologic type (**C**, **D**), IDH mutation (**E**, **F**) and 1p/19q col-deletion status (**G**, **H**). Log rank test was used to compare the difference between the two survival curves

Subgroup analysis showed that different subgroups of glioma patients with high FXYD2 mRNA expression also had longer OS. Among them, low-grade glioma (*P* = 0.011), high-grade glioma (*P* = 0.000), oligodendroglioma (*P* = 0.004), astrocytoma (*P* = 0.000), *IDH* mutant type (*P* = 0.000), *IDH* wild type (*P* = 0.180), *1p/19q* co-deletion type (*P* = 0.033), and *1p/19q* non-co-deletion type (*P* = 0.000) (Fig. 5).

### FXYD2 mRNA expression can predict the survival and prognosis of glioma patients in two independent databases

**Fig. 6:**
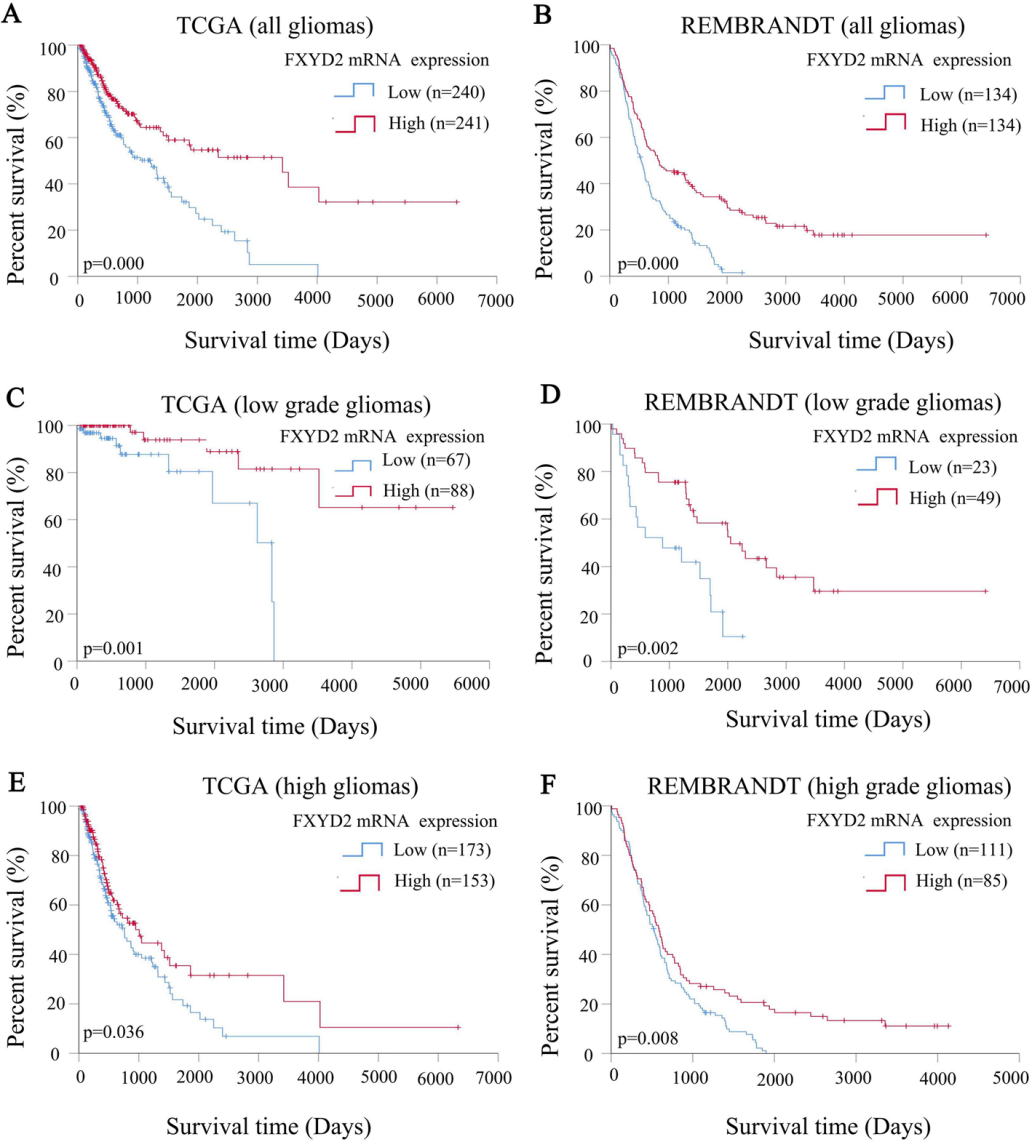
Kaplan–Meier curves for overall survival of different FXYD2 mRNA expression in TCGA and REMBRANDT databases. The Kaplan–Meier curves for overall survival of different FXYD2 mRNA expression in glioma patients with different WHO grade (**A**, **C**, **E**) in TCGA. The Kaplan–Meier curves for overall survival of different FXYD2 mRNA expression in glioma patients with different WHO grade (**B**, **D**, **F**) in REMBRANDT. Log rank test was used to compare the difference between the two survival curves

Using Kaplan–Meier survival curves, it was confirmed in two independent databases that glioma patients with high FXYD2 mRNA expression had a longer survival time than patients with low expression from TCGA (*P* = 0.000, Fig. 6A) and REMBRANDT database (*P* = 0.000, Fig. 6B). Further subgroup analysis on the two independent datasets showed that patients with low- or high-grade gliomas that had high FXYD2 mRNA expression also exhibited longer survival times (Fig. 6C–F).

### FXYD2 mRNA expression is an independent factor affecting the survival of glioma patients

Univariate Cox analysis was used to identify the factors affecting the survival of glioma patients, including sex, age, recurrence, histopathology (HE), WHO grade, *IDH* mutation status, *1p/19q* co-deletion status, methylation status of MGMT, extent of resection, radiotherapy or chemotherapy status, and FXYD2 mRNA expression. Multivariate Cox analysis showed that high FXYD2 mRNA expression (HR: 0.744, 95%CI 0.577–0.960, *P* = 0.023), *IDH* mutation (HR: 0.740, 95% CI: 0.549–0.997, *P* = 0.048), *1p/19q* co-deletion (HR: 0.389, 95% CI: 0.260–0.583, *P* = 0.000), and chemotherapy status (HR: 0.607, 95% CI: 0.446–0.825, *P* = 0.001) were independent factors associated with longer patient survival. Meanwhile, age (HR: 1.015, 95% CI: 1.006–1.024, *P* = 0.002), relapse (HR: 2.137, 95% CI: 1.692–2.698, *P* = 0.000), and WHO grade (HR: 2.700, 95% CI: 1.930–3.779, *P* = 0.000) represented independent factors associated with poor survival (Table 2).

**Table 2:** Correlation analysis between FXYD2 mRNA expression and overall survival among 516 glioma patients in CGGA

Parameter	Univariate Cox Regression			Multivariate Cox Regression		
	*P* value	HR	95% CI (low-up)	*P* value	HR	95% CI (low-up)
Sex	0.447	1.091	0.871–1.366			
Age	0.000	1.027	1.017–1.036	0.002	1.015	1.005–1.024
Recurrent	0.000	2.117	1.690–2.651	0.000	2.118	1.672–2.684
Histopathological types (HE)	0.000	1.827	1.657–2.015	0.333	0.896	0.719–1.118
2021 WHO glade	0.000	2.804	2.377–3.308	0.000	2.717	1.914–3.856
IDH mutation status	0.000	0.321	0.256–0.403	0.025	0.712	0.529–0.958
1p/19q codeletion status	0.000	0.282	0.199–0.400	0.000	0.403	0.265–0.611
MGMT methylation_status	0.004	0.790	0.674–0.926	0.041	0.841	0.713–0.993
Complete excision	0.713	1.050	0.809-1.364	0.201	0.838	0.639–1.099
Radiotherapy	0.467	1.109	0.839–1.465	0.244	0.825	0.598–1.140
Chemotherapy	0.230	1.176	0.903–1.533	0.003	0.611	0.443–0.841
FXYD2 mRNA expression	0.000	0.469	0.374–0.589	0.018	0.959	0.927–0.993

*HR* Hazard ratio, *CI* Confidence interval

### Increased FXYD2 mRNA expression can predict the chemosensitivity of glioma patients

According to the median expression of FXYD2 mRNA, the patients were divided into two groups: low or high expression. Univariate Cox analysis was used to investigate the related factors affecting the survival time of glioma patients, including sex, age, recurrence, histopathology, WHO grade, *IDH* mutation status, *1p/19q* co-deletion status, methylation status of MGMT, extent of resection, radiotherapy, and chemotherapy status. The results of multivariate Cox analysis showed that in the group with high FXYD2 mRNA expression, chemotherapy status (HR: 0.413, 95% CI: 0.254–0.671, *P* = 0.000), *IDH* mutation status (HR: 0.440, 95% CI 0.277–0.701, *P* = 0.001), methylation of MGMT (HR: 0.702, 95% CI 0.538–0.915, *P* = 0.009) and *1p/19q* co-deletion (HR: 0.420, 95% confidence interval 0.237–0.747, *P* = 0.003) were independent factors associated with longer survival. Meanwhile, relapse (HR value: 2.940, 95% CI: 1.995–4.332, *P* = 0.000), WHO grade (HR value: 3.799, 95% CI: 2.127–6.785, *P* = 0.000) were independent factors associated with poor survival (Table 3). However, in the group with low FXYD2 mRNA expression, chemotherapy status was not an independent factor affecting the survival of patients with glioma (univariate Cox analysis *P* = 0.132; multivariate Cox analysis *P* = 0.174; Table 3).

**Table 3:** Correlation analysis between chemotherapy and overall survival among glioma patients with high and low FXYD2 mRNA expression in CGGA

Parameter	Univariate Cox Regression			Multivariate Cox Regression		
	*P* value	HR	95% CI (low-up)	*P* value	HR	95% CI (low-up)
High						
Sex	0.002	1.792	1.231–2.607	0.123	1.396	0.914–2.132
Age	0.000	1.031	1.015–1.047	0.137	1.012	0.996–1.028
Recurrent	0.000	2.041	1.432–2.910	0.000	2.951	1.977–4.406
Histopathological types (HE)	0.000	2.037	1.754–2.365	0.415	0.861	0.601–1.233
2021 WHO grade	0.000	3.589	2.727–4.723	0.000	4.037	2.208–7.384
IDH mutation status	0.000	0.261	0.181–0.375	0.000	0.409	0.256–0.655
1p/19q codeletion status	0.000	0.309	0.199–0.480	0.004	0.426	0.237–0.765
MGMT methylation_status	0.021	0.729	0.558–0.952	0.015	0.710	0.539–0.935
Complete excision	0.669	1.092	0.730–1.634	0.678	0.913	0.594–1.403
Radiotherapy	0.256	1.300	0.827–2.043	0.468	0.816	0.472–1.413
Chemotherapy	0.909	1.024	0.682–1.537	0.001	0.427	0.259–0.704
Low						
Sex	0.101	0.786	0.589–1.048			
Age	0.000	1.023	1.011–1.035	0.024	1.014	1.002–1.026
Recurrent	0.000	1.880	1.399–2.525	0.000	1.859	1.360–2.540
Histopathological types (HE)	0.000	1.583	1.387–1.807	0.395	0.884	0.665–1.175
2021 WHO grade	0.000	2.222	1.818–2.716	0.000	2.416	1.564–3.730
IDH mutation status	0.000	0.472	0.347–0.642	0.730	0.933	0.628–1.385
1p/19q codeletion status	0.001	0.363	0.197–0.668	0.016	0.436	0.222–0.854
MGMT methylation_status	0.036	0.813	0.669–0.986	0.238	0.880	0.711–1.088
Complete excision	0.805	0.958	0.679–1.351	0.322	0.836	0.587–1.191
Radiotherapy	0.861	0.969	0.679–1.382	0.185	0.758	0.504–1.142
Chemotherapy	0.132	1.310	0.922–1.862	0.279	0.788	0.512–1.213

*HR* Hazard ratio, *CI* Confidence interval

## Discussion

The *FXYD2* gene is located on chromosome 11q23 [21], while the FXYD2 protein is the r subunit of the Na-K-ATP enzyme. FXYD2 has been shown to reduce the Na ion affinity of Na-K-ATP [22], resulting in subsequent inhibition of cell proliferation [23]. However, the expression and application value of FXYD2 mRNA in gliomas have not been previously reported.

This study revealed that the expression of FXYD2 mRNA is related to the degree of malignancy of gliomas. Specifically, higher degree malignancies are associated with lower FXYD2 mRNA expression, suggesting that FXYD2 mRNA expression can be used as a predictive biomarker for the degree of malignancy of gliomas. Moreover, FXYD2 mRNA expression was found to be related to the survival time of glioma patients with lower expression associated with shorter survival time, suggesting that it can also be used to predict patient survival prognosis. FXYD2 mRNA expression was also related to the chemosensitivity of glioma patients to TMZ. Meanwhile, TMZ represents an independent factor affecting the survival of glioma patients with high expression of FXYD2 mRNA, but not patients with low expression. Hence, we postulate that the expression of FXYD2 mRNA can be used to predict the chemosensitivity to TMZ. Specifically, patients with high FXYD2 mRNA expression will be more likely to respond to TMZ therapy, thereby prolonging survival time, while those with low expression will not benefit from this therapy. These results were similar to those reported by Hsu I-Ling et al. [24] who found that, compared with ovarian cancer cells expressing low levels of FXYD2, those with high expression were more sensitive to cardiosides, while cardiotonic glycosides can effectively inhibit the growth of ovarian cancer cells.

Jin et al. [25] found that FXYD family members are differentially expressed in colon cancer, which is a potential clinical biomarker of colon cancer and participates in the complex biological functions of tumor progression. The expression of FXYD2, FXYD3 and FXYD4 is an independent prognostic factor for for the survival of colon cancer. Currently, the underlying mechanism associated with the effects of FXYD2 in tumors is unclear. The Na-K-ATPase serves as the transport system for Na and K ions on the cell membrane [26], which serves to maintain the Na/K ion concentration gradient inside and outside of the cell. These gradients are essential for maintaining cell volume and membrane potential and also guarantee the maintenance of intracellular homeostasis [26]. They also provide nutrients to the cells and regulate the concentration of intracellular pH and calcium ions. Meanwhile, increased expression of FXYD2 was found to decrease the activity of Na/K-ATPase, Moreover, another study has reported that the body senses mechanical pain abnormalities caused by peripheral inflammation through FXYD2 in neurons [27]. Following peripheral tissue inflammation, the interaction between FXYD2 and the α subunit of Na/K-ATPase is enhanced, causing downregulation of Na/K-ATPase activity, while increasing neuronal membrane potential depolarization and excitability. The body then senses peripheral inflammatory stimulation signals, resulting in corresponding inflammatory stress and clearance responses.

## Conclusions

This study revealed that the expression of FXYD2 mRNA in gliomas can predict the degree of malignancy and survival time of patients. At the same time, FXYD2 mRNA expression can predict the chemosensitivity of glioma patients to TMZ. However, considering that our study is limited to mRNA, the transcriptional regulation, protein translation, as well as underlying regulatory mechanisms and pathways of FXYD2 remain unclear and require further investigation.

## Data Availability

The CGGA data used and analyzed during the current study are available from http://www.cgga.org.cn/download.jsp. The TCGA and REMBRANDT datasets used and analysed during the current study are available from http://www.cgga.org.cn/download_other.jsp. Public access to the databases is open.

## Abbreviations

FXYD2: Sodium/potassium-transporting ATPase subunit gamma
OS: Overall survival
WHO: World Health Organization
IDH: Isocitrate dehydrogenase
TMZ: Temozolomide
CCC: Clear cell carcinoma
CGGA: The Chinese glioma Genome Map Project
TCGA: The cancer genome map database
REMBRANDT: The molecular brain tumor database
MGMT: Methylation of O6 methylguanine DNA methyltransferase
HR: Hazard ratio
CI: Confidence interval
NEC: Not Elsewhere Classified
